# Mitigating the impacts of street lighting on biodiversity and ecosystem functioning

**DOI:** 10.1098/rstb.2022.0355

**Published:** 2023-12-18

**Authors:** Darren M. Evans

**Affiliations:** School of Natural and Environmental Sciences, Newcastle University, King's Road, Newcastle upon Tyne NE1 7RU, UK

**Keywords:** ALAN, moths, pollination, network ecology

## Abstract

Street lights are not only a major source of direct light pollution emissions, but stock has been transitioning to light-emitting diode (LED) technology in many parts of the world, resulting in increases in the blue part of the visible spectrum that is more harmful to biodiversity and human health. But LEDs can be modified more easily than conventional sodium lamps by adjusting their intensity, spectral output and other features of street light systems. In this Opinion piece, I provide an updated overview of street light mitigation strategies and contend that research in this area has been slow. I show how experimental lighting rigs that mimic real street lights can be used for mitigation testing, since invertebrate behaviour, abundances and interactions can respond quickly and measurably. I demonstrate how advances in network ecology that use species interaction data can provide much-needed assessments of the impacts of street lights on biodiversity and ecosystem functioning, and ultimately provide new tools and metrics for biomonitoring. I acknowledge the limitations of measuring local, short-term responses of biodiversity and identify promising avenues for collaborating with industry and government agencies in new or existing road lighting schemes, to minimize the negative long-term impacts at marginal cost.

This article is part of the theme issue ‘Light pollution in complex ecological systems’.

## 1. Introduction

Over the past decade, a burgeoning number of studies have revealed that night-time light pollution is not only increasing, but is having significant impacts on biological phenomena, from individual physiology and behaviour to communities and ecosystem functioning [1]. A wide variety of lighting devices contribute to this pollution, including street lighting, vehicles, advertising boards, commercial buildings and domestic sources. Of these, street lights are a major focus of attention as they are often the most persistent, aggregated and intense source of lighting in urban areas [2]. Furthermore, many parts of the world have been transitioning their lighting stock from narrow-spectrum (e.g. high-pressure sodium, HPS) to ‘broad white’ spectrum (light-emitting diode, LED) lamps, resulting in increased emissions in the blue part of the visible spectrum [1,3]. Indeed, using photographs taken by astronauts onboard the International Space Station, Sánchez de Miguel *et al.* [1] recently showed that these spectral changes have been widespread across Europe over the past 10 years, and that there has been a pronounced whitening of artificial light at night (ALAN). While the benefits of LED street lights may seem obvious in terms of improved energy efficiency, it has long been known that many biological phenomena (e.g. melatonin production) are spectrally dependent on and/or sensitive to blue emissions [4]. Recently, Boyes *et al*. [5] not only demonstrated the direct impacts of street lights on local insect populations, but also that negative effects were more pronounced under white LED lamps compared to HPS. Thus, the combined effects of ALAN increasing globally [6] and the transition from narrow- to broad-spectrum street lighting [1] will have substantial consequences for biodiversity and ecosystem processes. It is clear that a very different approach to the use of artificial lighting is required [4].

In this paper, I provide an overview of the mitigation methods to minimize the impacts of ALAN on biodiversity (and human health), acknowledging that these have been considered for some time [2,7] but nevertheless remain a major challenge. I then give my opinion as to how (i) street light mitigation options can be quickly and efficiently tested in the field; and (ii) advances in ecological network construction methods and analyses provide a framework in which the responses of entire communities of interacting communities, and the ecological processes they provide, can be assessed. I discuss how this could ultimately lead to new biomonitoring metrics for policy makers and land managers. Although the impacts of skyglow on biodiversity is an important area of research [7], here I specifically focus on the impacts of street lights (as a major source of direct emissions) on terrestrial plant–invertebrate interactions, especially the consequences of the increasingly widespread use of LED technology both for new lighting installations and to retrofit existing systems. Finally, I identify priorities for street light mitigation testing and validation using adaptable experimental rigs.

## 2. Impacts of street lights on biodiversity and ecological processes

A number of qualitative reviews have examined the impacts of ALAN on a range of taxa in different habitats [8–10], underlining just how widespread these effects are, as well as the current knowledge gaps [11]. Sanders *et al.* [12] conducted a meta-analysis of the biological impacts of ALAN, with results showing how it changes: (i) the physiology and behaviour of organisms (by affecting hormone levels); (ii) the onset of daily activity; (iii) feeding; and (iv) phototaxis. However, they found a weaker impact on community responses, such as abundance and species richness, and suggested that the impacts of ALAN on community structure and diversity are less clear, and could depend on the impacts on key species or groups [13].

One area examining community responses to ALAN considers species' visual systems and how they differ in their sensitivity to different wavelengths of light [14]. Longcore *et al.* [15] used species response curves to predict which configurations of a customizable LED system would attract fewer insects and showed a significant decrease in insect attraction for the custom spectra selected on this basis. Longcore *et al.* [16] then formalized a strategy to use spectral response curves for species and spectral power distributions from lamps in order to assess potential impacts by comparing the relative effect of additional lux from the lamp with an additional lux of daylight. While this approach shows promise for mitigating the impacts of street lights on whole communities of species, predicting biological responses to different lamp technology and lighting strategies is still hampered by the current lack of readily available response curves across taxonomic groups. Gaining more complete data on species response curves is thus a research priority.

**Figure 1.**
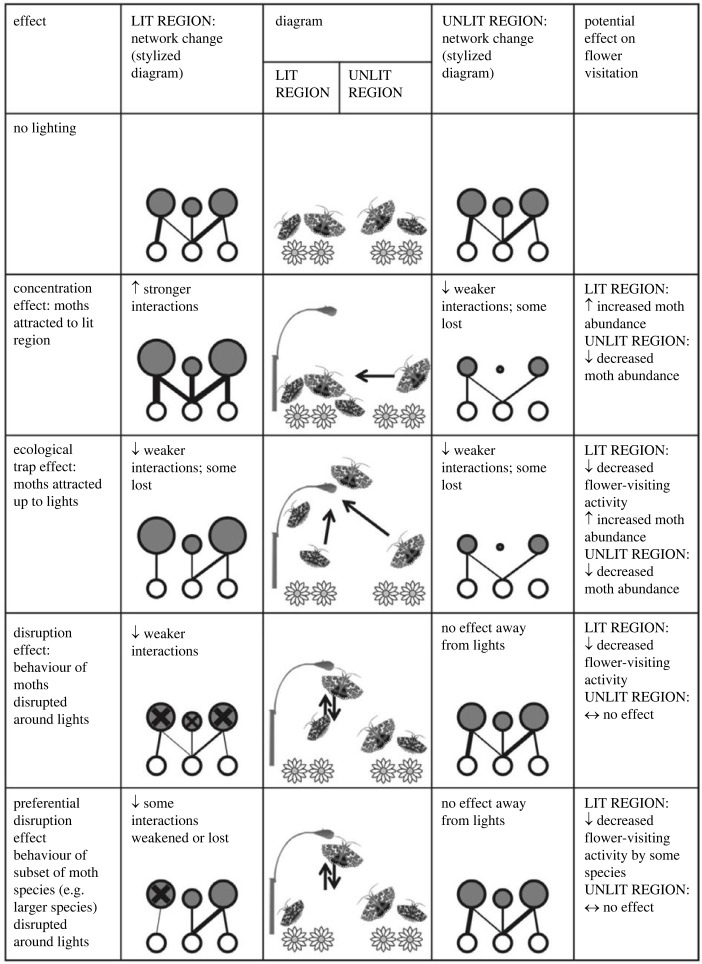
Possible scenarios for change in plant–moth pollination networks as a result of artificial night lighting, with predictions for effects on local flower-visitation activity by moths (with permission from Macgregor *et al.* [20]). In network representations, nodes represent species (lower = flowering plants, upper = moths) and links represent pollination interactions. Node size represents relative species abundance and link thickness represents interaction strength. Crosses indicate disruption of behaviour.

Many studies investigate the impacts of ALAN on species, populations and communities, but few to date have focused on the impacts on ecosystem functioning [12]. This is surprising given, for example, the increasing recognition that nocturnal moths are important pollinators [17–19]. In a review, Macgregor *et al.* [20] identified that moths are not only important pollinators of a diverse range of plant species in various ecosystems across the world, but that the ecosystem services they provide could be impacted by ALAN. Possible scenarios for disruption to pollination by moths (figure 1) include (i) a concentration effect, where moths are attracted to areas under street lights; (ii) an ecological trap effect, where moths are attracted away from plants to lamps; (iii) a disruption effect driven by changes in moth behaviour around lights; or (iv) a preferential disruption effect where the behaviour of a subset of moth species is affected (for example based on species response curves as described above). They then went on to test for the first time the impacts of existing UK street lights on moths and their biotic interactions, using a matched-pair design of lit and unlit sites [21]. Moth abundance at ground level was halved at lit sites, species richness was more than 25% lower, and flight activity at the level of the light was 70% greater. To assess the quantity and diversity of pollen transported by moths (an important ecological process [22]), all moths retained during sampling were examined for pollen. Of these 23% were found to be carrying pollen, and from a high diversity of plant species, but crucially the study revealed an overall reduction in pollen transport at lit sites, suggesting that street lighting potentially impacts on pollination by nocturnal invertebrates. Further experimental work has since examined the effects of street lighting on moth populations [5,23] and plant–pollinator interactions [24]. The degree to which this work is generalizable to other measures of ecosystem functioning is unclear, but examining the consequences of altered species interactions in above- and below-ground communities is clearly an important area of research.

I discuss next how advances in network ecology, a unifying framework for reconciling the structure and function of biodiversity [25], can be used to assess the potential impacts of street lighting on entire communities of interacting organisms, and on the ecological processes they perform.

## 3. Network ecology for assessing the impacts of street lights on biodiversity and ecosystem functioning

There have been significant advances in the theoretical understanding, construction, analysis and application of complex species interaction networks in recent years [26–30]. Ecological networks describe the interactions between species, the underlying structure of communities and the function and stability of ecosystems [31]. They have the potential to quantify the effects of human activities on a wide range of ecological interactions [32–35] and have the potential to provide new metrics for biomonitoring by providing information on changes to network structure and complexity. It has long been argued that measures of network nestedness and connectance could easily be incorporated into biomonitoring programmes [36]. Indeed, classical studies in network ecology have demonstrated that conventional community indices (e.g. species richness) can fail to discriminate the impacts of environmental change, indicating that perturbation of the structure and function of ecological communities might be overlooked in studies that do not quantify species interactions [37].

Using the example of nocturnal moths as pollinators, plant–pollinator network construction methods can be either plant- or insect-focussed (but preferably both) [29]. Macgregor *et al.* [38] demonstrated how DNA metabarcoding of moth pollen loads can be successfully used to construct networks, in addition to the conventional sampling method of catching moths visiting flowers [21]. Banza *et al.* [18] examined the extinction dynamics of moth pollen transport networks, then went on to examine the impacts of wildfire on network structure and complexity [39], and, using network ‘robustness’ analyses, concluded that if wildfires become more frequent as a result of climate change, community resilience may be eroded. Returning to the possible effects of street lights on plant–moth pollinator interactions, Macgregor *et al.* [20] provided a comprehensive overview of how ecological networks could reveal changes in abundances and interaction strengths. For example, under lights, an ecological trap effect could be detected as increased moth abundance but a decrease in flower-visiting activity was observed, with potentially some interactions completely lost (figure 1). This is a very good conceptual framework for mitigation hypothesis forming and testing.

Using an ecological network approach, Knop *et al.* [40] showed that ALAN disrupts nocturnal pollination networks (with the potential to cascade from the nocturnal to diurnal pollinator community) and that this has negative consequences for plant reproductive success. Mobile street lamps were erected in light-naive meadows in Switzerland, and nocturnal interactions between plants and flower visitors were compared in lit and unlit areas. They found a significantly lower number of flower visits (62%) and flower-visiting species (29%) at illuminated sites. Network nestedness (i.e. the degree to which species with few links have a subset of the links of other species, rather than a different set of links) was significantly higher in dark sites, suggesting that ALAN might have a destabilizing effect on nocturnal plant–pollinator networks. The consequence of altered network structure was assessed by examining the reproductive output of a plant bioassay (*Cirsium oleraceum*) with a 13% reduction in fruit set observed, even though the plant also received numerous visits from diurnal pollinators. Macgregor *et al.* [24] conducted a similar experiment but using a different plant bioassay (*Silene latifolia*) and found contrasting effects. This suggests that some plants might be ‘winners’ and others ‘losers’ under lit conditions; examining the responses of plant communities should thus warrant further attention. Nevertheless, the study by Knop *et al.* [40] suggests that examining the responses of entire communities of interacting species to the impacts of street lights is not only possible, but preferable given that indirect and cascading effects are shown to be important. Moreover, the use of experimental lighting rigs, combined with ecological network analyses, can play a key role in street light mitigation studies by examining the response of species abundances, interactions and ultimately ecological processes and ecosystem functioning.

## 4. Street light mitigation strategies

Protecting (and expanding) natural unlit areas is likely to be the most effective option for reducing the ecological effects of lighting. However, this could conflict with other social and economic objectives. According to Gaston & de Miguel [4], the conventional sequence of mitigation hierarchy elements, in order of progressively reducing desirability, is: avoid, minimize, restore or rehabilitate, then offset. In the case of ALAN, however, they argue that the hierarchy should probably be: avoid, restore or rehabilitate, minimize and then offset. Methods to minimize the impacts of ALAN on biodiversity and human health are not new [2,41] but nevertheless remain a major challenge. Indeed, there are few light mitigation studies in the literature relative to the burgeoning number showing adverse effects of ALAN on a range of taxa. Street lighting is usually required to illuminate the road surface and objects below the level of the light source, but poor design can lead to significant proportions of the light being emitted either upwards or horizontally [2]. In the UK, a number of local authorities either dim existing street lights, or switch them off altogether late in the night for energy saving reasons and/or to reduce the impacts on biodiversity. But surprisingly few studies have been conducted to determine whether the latter is effective.

Specific street light mitigation measures are covered in detail elsewhere (e.g. [2]) and include: (i) part-night lighting, where lamps are switched off during periods of low need (e.g. after midnight), are seasonally adaptive or are user-responsive (e.g. motion-sensitive); (ii) shielding/trespass to prevent light overspill beyond the intended purpose; (iii) minimizing the number of street lights in a given area; (iv) off-setting; (v) dimming; and (vi) spectral manipulation. The latter is particularly pertinent in the context of the transition of street lighting stock in many parts of the world to LED lamps. Due to differences in colour and intensity, and other characteristics like flicker and non-Lambertian emission, LEDs have a different, more profound effect on wildlife than past lighting models [14], disrupting the balance of species interactions [42] and creating unprecedented niche overlaps between nocturnal and diurnal species [20]. However, they can readily be modified by reducing intensity and/or duration, controlling spill or manipulating spectrum to avoid the peak sensitivities of most animal groups to shorter wavelengths. Thus, there is considerable scope for multiple combinations of experimental street light treatments to determine the least damaging arrangements for biodiversity and ecosystem functioning.

## 5. Mitigation testing using experimental lighting rigs

**Figure 2.**
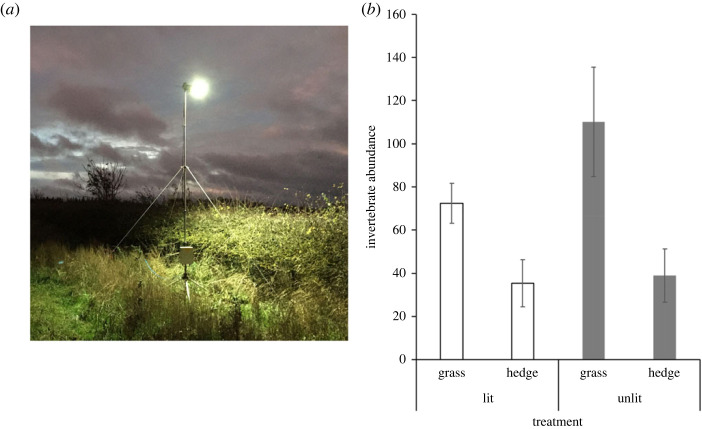
(*a*) An example of an experimental lighting rig fitted with an industry-standard lamp at 4 m height at Cockle Park Farm, Newcastle University, UK, with adjacent grass margin and hedgerow habitat. (*b*) Preliminary results of mean invertebrate abundances sampled under paired (*n* = 9) lit (white) and unlit (grey) treatments in Oct–Nov 2021, from grass and hedgerow habitats using 14 m transects (see §5). (Online version in colour.)

Over the past decade, innovative experimental designs have been used to determine the impacts of street lights on plants, moths, bats and a range of other taxa [24,40,43–45]. In particular, mobile, experimental lighting rigs, which are designed in collaboration with industry specialists to mimic conventional street lights, have been used to study a range of plant and animal responses. These usually consist of industry-standard lamps attached to bespoke, telescopic tripods (figure 2) that could be fitted with timers and/or sensors to control when the lamps switch on/off. In the UK, 4 metre lighting rigs have been used as this is the minimum height for highway lighting and a common height for street lighting on minor roads in rural settings. Crucially, mobile rigs can be positioned in light-naive environments, removing the confounding effects of background light pollution (skyglow) from experiments [5]. Positioning generators well away from the lights is also an established protocol [44].

Stone *et al.* [43,44] used experimental lighting rigs to demonstrate the impacts of street lighting on commuting bats, and Macgreor *et al.* [24] used them to show impacts on a nocturnally pollinated plant. Of particular interest for mitigation studies is a recent short-term experiment on caterpillar feeding behaviour by Boyes *et al.* [5]. Here, lighting rigs were set up along homogeneous, previously unlit grass field margins 1 h before sunset in SE England. Sampling was then conducted between 1 and 2 h after dusk to test whether ALAN disrupted the normal feeding behaviour of nocturnal caterpillars. In the context of a wider study examining the impacts of street lighting on the larval stages of moths, they found significant differences in caterpillar abundance between lit and unlit areas, with fewer caterpillars sampled by sweep netting under white LED light (there was no statistically significant difference under HPS lights). Although the rigs were used in this study to test whether ALAN disrupted the normal feeding behaviour of nocturnal caterpillars, it is intriguing that statistically significant effects of lighting on this insect group could be observed after only a few days.

In order to investigate whether lighting could affect a broad range of arthropods (beyond moth caterpillars), I conducted a field experiment using a matched-pair design of nine lit and unlit grass margin sites at Cockle Park Farm, Newcastle University between 13 October and 26 November 2021. Briefly, experimental rigs described in [24] were fitted with an LED light and illuminated for two consecutive nights. In the morning following the second night, hedgerow beating along a 14 m transect (at 0, 7 and 14 m points) and grass sweep netting were carried out in the paired sites (*n* = 9), broadly following [5], with arthropods subsequently processed, counted and identified in the laboratory (total = 2313). Preliminary results from this pilot study show a statistically significant effect of the lighting treatment on arthropod abundance (across 11 orders), with approximately 35% fewer found in lit sites compared to unlit sites (except Araneae, which showed an increase), and with the biggest differences in grass habitats (figure 2). Although late Autumn is not the ideal time to sample arthropods (most leaves from the hedge had dropped), the preliminary results of the study are important for two reasons. First, it shows that broad (mostly grassland) invertebrate assemblages can be adversely affected by street lighting, at least in the short term, contrary to a previous study that used pitfall traps with light 1 m above the ground [46]. Second, the study demonstrates the potential of experimental lighting rigs for mitigation testing, as invertebrate assemblages appear to respond very quickly to two nights of street light illumination, making meaningful comparisons of different mitigation strategies possible in a short timeframe, although more research is necessary.

One immediate application of experimental lighting rigs for mitigation testing is to compare the impacts of different lamp technologies on invertebrate communities. For example, Boyes *et al.* [5] sampled significantly fewer caterpillars by sweep netting under white LED light compared to unlit but found no statistically significant differences under HPS lights compared to unlit. Testing the (at least short term) effects of LED spectral manipulation (custom colours and filters), dimming (especially of LED) and shielding (to direct/control lighting and prevent glare) on invertebrate assemblages is feasible using this experimental set-up and should be a research priority. The rigs shown in figure 2 are fitted with sensors, enabling the lights to automatically illuminate at dusk and switch off at dawn, as well as with timers for specific switch on/off times, making it possible to test for the effects of part-night lighting (e.g. 00 : 00 switch off (e.g. [24])). They can also be easily modified to allow for dimming, for user-responsive lighting or for combinations of treatments. Moreover, the development of automated biomonitoring technology means that rigs could soon be fitted with sensors to examine the responses of a broad range of taxa [47]. However, while all this shows promise for street light mitigation testing, it should be stressed that only the short-term responses of biodiversity are observed, and therefore more detailed studies of the responses of plant–animal interactions and population dynamics under street lights are essential [5,20,24,48].

## 6. Scaling up street light mitigation studies

I have shown how experimental street light rigs have been successfully used to examine the responses of species, populations, communities and, more recently, quantitative plant–pollinator networks to ALAN. While a plethora of plant–pollinator network studies have helped to provide a better understanding of mutualistic networks in space and time [49], state-of-the-art approaches in network ecology merge a range of interaction types, across trophic levels, into multilayer networks [26]. These can also include socio-ecological information [50]. For example, it is possible to use DNA/RNA-based methods to construct plant–caterpillar–parasitoid networks [28] or even below-ground plant–microbe networks (including viruses) [51], with new analytical methods available for examining the impacts of disturbance [35,52], such as ALAN. Thus, the effects of street light mitigation on biodiversity and ecosystem functioning can be examined in a much more holistic and informative manner using network approaches. For bipartite networks in particular, incorporating network attributes into biomonitoring surveys is relatively easy [53] and networks constructed using molecular methods also allow eco-evolutionary responses to be examined [36]. Nevertheless, more research is needed to understand the effects of street lights at the base of the food chain, and from this, the indirect impacts of lighting on higher taxa through networks of ecological interactions can be examined [5].

While my focus has been on the use of experimental rigs for mitigation testing, it is clear that this will only provide information in the short term; the long-term impacts on biodiversity and ecosystem functioning across large spatial scales remains poorly understood. For example, recent work by Brown *et al.* [54] showing arthropod responses to newly introduced light over a short timeframe is likely a result of phototaxis, whereas Davies *et al.* [46] exposed communities to light over multiple years, thereby demonstrating a systematic impact. Indeed, a major study on macro-moth communities showed that the negative impact of different light spectra became evident only after 2 years of exposure [23]. Furthermore, the effects of skyglow on species interactions and network structure have yet to be fully explored.

By assuming all UK major roads have street lights, one study estimated that these affect 3.2% of the UK's total land area at over 1 lux [55]. Boyes *et al.* [5] estimated that just 1.1% of the land area encompassing their study sites in SE England was directly illuminated to this level and concluded that the effect of direct illumination by street lights has probably been a minor contributor to long-term national moth declines to date. However, the impacts on broad-scale ecosystem service provision by moths are unclear. There are useful mapping tools for modelling the night-time light environment at a high spatial resolution in urban areas for further ecological investigations (e.g. [56]), but more research is needed at larger spatial scales. Longcore [14] provides a methodology for creating maps to assess and mitigate the impacts of lighting on sensitive species, highlighting the need for innovation that integrates geographic information systems (GIS) with lighting calculations (that could include different lamp types and shielding mitigation methods, for example) in a way that can be deployed over large geographical areas. Although in early development, their results demonstrate a workable approach for calculating the impacts of new road lighting schemes introduced into the habitats of sensitive species.

## 7. Conclusion

In this Opinion piece, I have re-asserted the need for more street lighting mitigation studies and demonstrate how lighting-rig experiments, combined with ecological network analysis, can determine the effects of street lighting on biodiversity and ecosystem functioning, at least in the short term. Thus, there is considerable scope for modifying new and existing street lights to be less harmful to biodiversity and human health. With stock transitioned/transitioning from narrow to broad white LED spectrum lamps in many parts of the world, there are clear opportunities for mitigation as LEDs can be modified more easily than sodium lamps by adjusting their intensity (dimming) and spectral output (custom colours and filters) [15,57]. Given that I have shown that invertebrates respond rapidly (both in terms of behaviour and abundance) to the introduction of experimental lighting rigs in light-naive environments, there is a clear pathway to test a combination of LED mitigation measures (e.g. spectral manipulation, dimming, shielding and part-night lighting) on plant–animal interactions [24]. This could also be expanded to domestic lighting (e.g. [58]). Furthermore, ecological network approaches can provide potential new metrics for biomonitoring [36], although this area is underdeveloped. The next challenge will be to scale-up local, short-term results to the landscape scale, but recent work shows promise, particularly if conducted in collaboration with industry and government agencies [14], taking into account the needs of local communities. Finally, emerging biomonitoring sensors (e.g. fitted to new and existing street lights) and other technologies (e.g. eDNA) provide new opportunities for assessing broad-scale mitigation strategies.

## Data Availability

The data used in the preliminary analysis are provided in the electronic supplementary material [59].
